# Plasma levels of anti phosphocholine IgM antibodies are negatively correlated with bone mineral density in humans

**DOI:** 10.1038/s41598-025-85624-9

**Published:** 2025-01-15

**Authors:** Michela Palmieri, Spyridoula Maraka, Horace J. Spencer, Jeff D. Thostenson, Katherine Dishongh, Micheal Knox, Betty Ussery, Jesse Byrd, Jacqueline K. Kuipers, Sanaz Abedzadeh-Anaraki, Chitharanjan Duvoor, Yuanjie Mao, Lakshmi Menon, James S. Williams, Stavros C. Manolagas, Robert L. Jilka, Elena Ambrogini

**Affiliations:** 1 Division of Endocrinology and Metabolism and Center for Musculoskeletal Disease Research, University of Arkansas for Medical Sciences, 4301 W. Markham, #587, Little Rock, AR 72205 USA; 2 Central Arkansas Veterans Healthcare System, Little Rock, AR USA; 3 Department of Biostatistics, University of Arkansas for Medical Sciences, Little Rock, AR USA; 4 Richmond Hill Medical Clinic, Richmond Hill, ON Canada; 5 St Bernards Medical Center, Jonesboro, AR USA; 6 Diabetes Institute, Ohio University, Athens, OH USA

**Keywords:** Oxidized phospholipids, Anti-PC IgM antibodies, Bone mineral density, Endocrinology, Endocrine system and metabolic diseases

## Abstract

Phosphatidylcholine is a ubiquitous phospholipid. It contains a phosphocholine (PC) headgroup and polyunsaturated fatty acids that, when oxidized, form reactive oxidized phospholipids (PC-OxPLs). PC-OxPLs are pathogenic in multiple diseases and neutralized by anti-PC IgM antibodies. The levels of anti-PC IgM increase as the levels of PC-OxPLs increase and, in humans, are inversely correlated with the incidence of cardiovascular diseases and steatohepatitis. PC-OxPLs also decrease bone mass in mice. Overexpression of anti-PC IgM ameliorates atherosclerosis and steatohepatitis, increases bone mass in young mice, and protects against high fat diet- and age-associated osteoporosis. We investigated the relationship between anti-PC IgM plasma levels and bone mineral density (BMD) in a cross-sectional study of 247 participants [mean age: 65.5 (± 8.6) years] without medical conditions known to influence BMD or antibody production. Anti-PC IgM levels negatively correlated with both T- and Z-scores at the lumbar spine, femur and, to a lesser extent, the forearm. These correlations were maintained after adjustment for age, race, and sex. These results raise the possibility that higher levels of anti-PC IgM in patients with lower BMD reflect exposure to higher levels of PC-OxPLs, which are known to affect bone mass, and could be a novel risk marker for osteoporosis.

**Supplementary Information:**

The online version contains supplementary material available at 10.1038/s41598-025-85624-9.

## Introduction

Phosphatidylcholine is the most ubiquitous phospholipid in cell membranes, microvesicles, and lipoproteins. It contains a phosphocholine (PC) head group and polyunsaturated fatty acids (PUFA) that are susceptible to enzymatic and non-enzymatic peroxidation leading to the formation of highly reactive oxidized phospholipids (OxPLs)^1^. OxPLs are generated during oxidation of low-density lipoproteins (LDL) and during cell apoptosis^1^, and their levels increase with age^2,3^. Unless neutralized or eliminated, OxPLs are pro-inflammatory and can be highly toxic^1,4^. Extensive evidence from murine models and humans has revealed that OxPLs with a PC head group (PC-OxPLs) are pathogenic in several diseases, including, atherosclerosis^1,5,6^, ischemia–reperfusion injury^7,8^, steatohepatitis^9,10^, macular degeneration^11^, multiple sclerosis^12^, inflammatory pain, osteoarthritis^13^ and osteoporosis^14–16^.

Following the peroxidation of PUFA, the phosphocholine headgroup of OxPLs undergoes a conformational change and becomes one of the damage-associated molecular patterns (DAMPs) recognized and bound by receptors of the innate immune system. These receptors are either expressed on the cell surface, such as the scavenger receptors and toll-like receptors^17–21^, or are soluble such as anti-PC IgM antibodies that recognize the PC portion of OxPLs, but not the PC of native phospholipids^22^. Binding of PC-OxPLs to these receptors activates defenses designed to prevent cell damage. However, when PC-OxPLs are not neutralized and/or their production is excessive, relatively to the capacity of those defense mechanisms, they become pathogenic in multiple diseases^1^.

Anti-PC IgM produced by B1 lymphocytes are a component of the innate immune system; the antigen binding sites of these antibodies are generated by rearrangement of germline-encoded variable region genes in the complete absence of foreign antigen exposure. In mice, the E06 IgM (also known as T15) is already present in embryos, neutralizes the bioactivity of PC-OxPLs and facilitates their clearance by promoting their uptake by macrophages^23,24^. Increased levels of E06 IgM or transgenic expression of a single chain variable fragment of the E06 IgM antibody, called E06-scFv, protects against atherosclerosis^1,5,6^, ischemia–reperfusion injury^7,8^, steatohepatitis^9,10^, inflammatory pain and osteoarthritis^13^.

In earlier work of ours, we used E06-scFv transgenic mice as a model to study the effects of PC-OxPLs in bone. We reported that these mice were protected against high fat diet induced bone loss^14^. Moreover, E06-scFv increased trabecular and cortical bone mass in 6-month-old mice fed a normal diet^16^. The effect of E06-scFv was dose dependent, and the bone mass increase was greater in homozygous as compared to hemizygous mice. Additionally, E06-scFv attenuated the age-associated bone loss in both female and male homozygous mice that were maintained on a normal diet and aged up to 22–24 months^15^. E06-scFv exerted this protective effect mainly by increasing osteoblastic bone formation. Collectively, these results, indicate that PC-OxPLs are important pathogenic factors in bone and their effect results from the suppression of bone formation.

Humans do not have the murine equivalent of T15 anti-PC^25^. They do, however, have anti-PC IgM, which are low at birth and slowly increase in the first two years of age^26^, comprising eventually between 5 and 10% of the total IgM pool^27^. This evidence indicates that, in humans, anti-PC antibodies develop through a combination of both pre-programmed, genetically determined, innate immunity and acquired immunity after post-natal exposure to PC.

Multiple studies have evaluated the relationship between endogenous levels of anti-PC IgM and chronic inflammatory diseases in humans. Low levels of anti-PC IgM are a risk marker for atherosclerosis and cardiovascular disease, whereas higher levels of anti-PC IgM are associated with lower risk of atherosclerosis, cardiovascular disease and chronic inflammation^28^. However, in a large prospective study of patients presenting with acute coronary syndrome, higher anti-PC IgM levels were not associated with protection against cardiovascular diseases^29^.

Here, we sought to determine whether endogenous anti-PC IgM plasma levels correlate with bone mineral density (BMD) in a population of Veterans cared for at the Central Arkansas Veterans Healthcare System (CAVHS). We performed a cross-sectional study of 247 subjects and found that the levels of anti-PC IgM were negatively correlated with both the T- and Z-scores at femur, spine, and forearm. These data support the interrelated working hypotheses that: higher levels of endogenous anti-PC IgM in patients with lower BMD reflect exposure to higher levels of PC-OxPLs. This endogenous increase of anti-PC IgM levels is insufficient to confer protection against osteopenia or osteoporosis and, it could be an osteoporosis risk marker.

## Results

### Characteristics of study participants

We enrolled 251 patients. Four patients were excluded from the analysis because a blood draw could not be obtained in three of them and the sample collected from the 4th patient was compromised. Therefore, the analysis includes 247 participants, 140 female and 107 males, with a mean (± SD) age of 65.6 ± 8.6 years (range 33–98); 77.7% were White and 21.1% African Americans. The patients’ demographics, height, weight, body mass index, blood pressure measurements, and associated comorbidities are shown in Table 1. Hypertension, diabetes mellitus and hyperlipidemia were present in 70.9%, 36.4% and 89.1% of the patients, respectively.

**Table 1:** Demographic and clinical characteristics of study participants (n = 247).

Variable	Mean (SD)
Age (years)	
Age at blood collection	65.6 (8.6)
Age at DXA	65.5 (8.6)
Height (inches)	66.7 (4.1)
Weight (lbs)	189.4 (45.7)
Body mass index (kg/m^2^)	29.88 (6.63)
Systolic blood pressure (mm Hg)	136.7 (18.4)
Diastolic blood pressure (mm Hg)	80 (10.42)
Variable	% (n)
Sex	
Female	56.7 (140)
Male	43.3 (107)
Race	
White	77.7 (192)
African American	21.1 (52)
Other racial groups	1.2 (3)
Hypertension	70.9 (175)
Diabetes mellitus	36.4 (90)
Hyperlipidemia	89.1 (220)

SD, standard deviation; DXA, dual-photon x-ray absorptiometry; n, number.

Dual-photon x-ray absorptiometry (DXA) scan was obtained in all patients (Table 2). Lumbar spine measurements were not available in 26 patients because of one or more of the following reasons: body habitus, aortic calcifications, presence of aortic endograft, compression deformities, osteoarthritic changes, scoliosis, presence of surgical hardware or other surgical artifacts, and presence of nerve stimulator or vertebroplasty. DXA measurements at the femur could not be obtained in 8 patients because of bilateral hip replacement. Forearm measurements were available in all patients. As indicated in Table 3, at the lumbar spine, the majority of patients, 65.6%, had normal BMD (T score ≥ − 1), 25.3% had osteopenia (T-score between < − 1 and > − 2.5), and 9% had osteoporosis (T-score ≤ − 2.5). Similarly, at the femoral trochanter and total femur, 59–61% of patients had normal BMD, 33–35% had osteopenia, and 5–5.8% had osteoporosis. At the femoral neck, however, a higher number of patients, 51%, had osteopenia, while 39% had normal BMD and 9.6% had osteoporosis. At the forearm, the majority of patients had normal bone density, 19–37% had osteopenia, and 9–14.9% had osteoporosis. The summary of laboratory measures available for the enrolled patients is shown in Supplementary Table S1.

**Table 2:** DXA BMD, T- and Z-scores scores summary.

Site	N	BMD mean (SD)	T-score mean (SD)	Z-score mean (SD)
Vertebra (L1–L4)	221	1.026 (0.165)	− 0.51 (1.48)	0.86 (1.54)
Femur				
Neck	239	0.752 (0.135)	− 1.22 (1.01)	0.09 (0.99)
Trochanter	239	0.665 (0.153)	− 0.70 (1.06)	0.11 (1.07)
Total	239	0.901 (0.153)	− 0.73 (1.03)	0.22 (1.02)
Forearm				
Diaphysis	247	0.721 (0.102)	− 0.47 (1.42)	1.03 (1.45)
Ultradistal	247	0.427 (0.078)	− 1.04 (1.28)	0.17 (1.25)
Total	247	0.481 (0.092)	− 0.88 (1.41)	0.52 (1.44)

**Table 3:** Percentage of patients with normal T-score, osteopenia or osteoporosis.

Site	N	Normal T score ≥ − 1% (n)	Osteopenia T-score < − 1 to > − 2.5% (n)	Osteoporosis T-score ≤ − 2.5% (n)
Vertebra (L1–L4)	221	65.61% (145)	25.34% (56)	9.05% (20)
Femur				
Neck	239	39.33% (94)	51.05% (122)	9.62% (23)
Trochanter	239	59.83% (143)	35.15% (84)	5.02% (12)
Total	239	61.09% (146)	33.05% (79)	5.86% (14)
Forearm				
Diaphysis	247	70.85% (175)	19.84% (49)	9.31% (23)
Ultradistal	247	48.99% (121)	37.65% (93)	13.36% (33)
Total	247	59.11% (146)	25.91% (64)	14.98% (37)

### Correlations of anti-PC IgM levels and BMD

Plasma levels of anti-PC IgM were measured by ELISA. Anti-PC IgM were log-transformed, and the correlation with BMD parameters was analyzed by Spearman’s correlation coefficients. Anti-PC IgM correlated with total IgM (r = 0.58, *p* < 0.0001) (Supplementary Table S2), and did not differ by sex or race (Supplementary Fig. S1a,b). The anti-PC IgM levels did not correlate with age (Supplementary Fig. S1c).

**Fig. 1:**
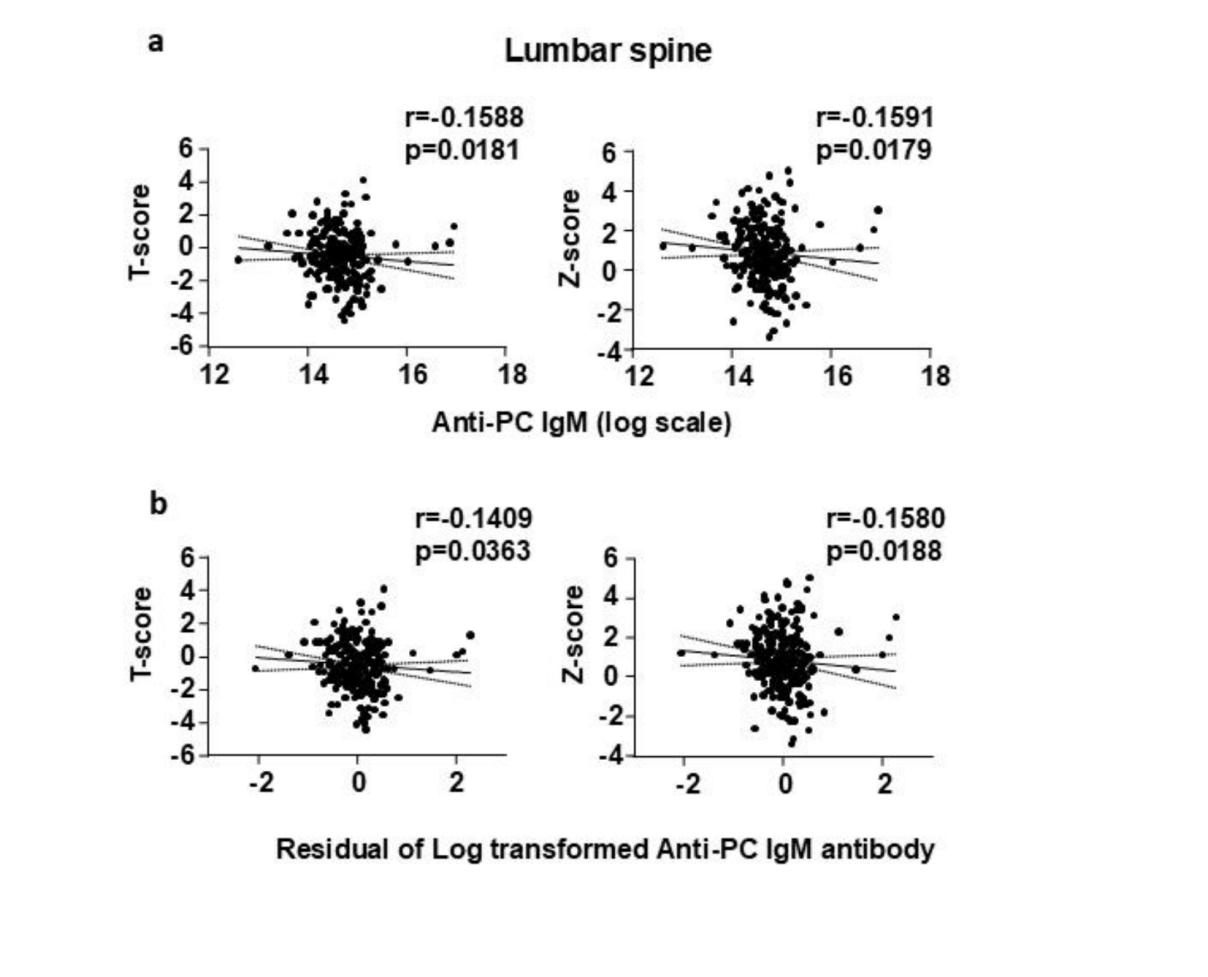
(**A**) Spearman correlation coefficients assessing the linear association between anti-PC IgM antibodies levels and DXA T-scores and Z scores at the lumbar spine. (**B**) Spearman correlation coefficients assessing the linear association between age-, sex-, and race-adjusted anti-PC IgM antibodies and DXA T-scores and Z-scores at the lumbar spine. A logarithmic transformation was applied to the anti-PC IgM measures prior to analysis. A linear regression on anti-PC IgM measures with covariates of age, sex, and race was used to produce the residuals.

**Fig. 2:**
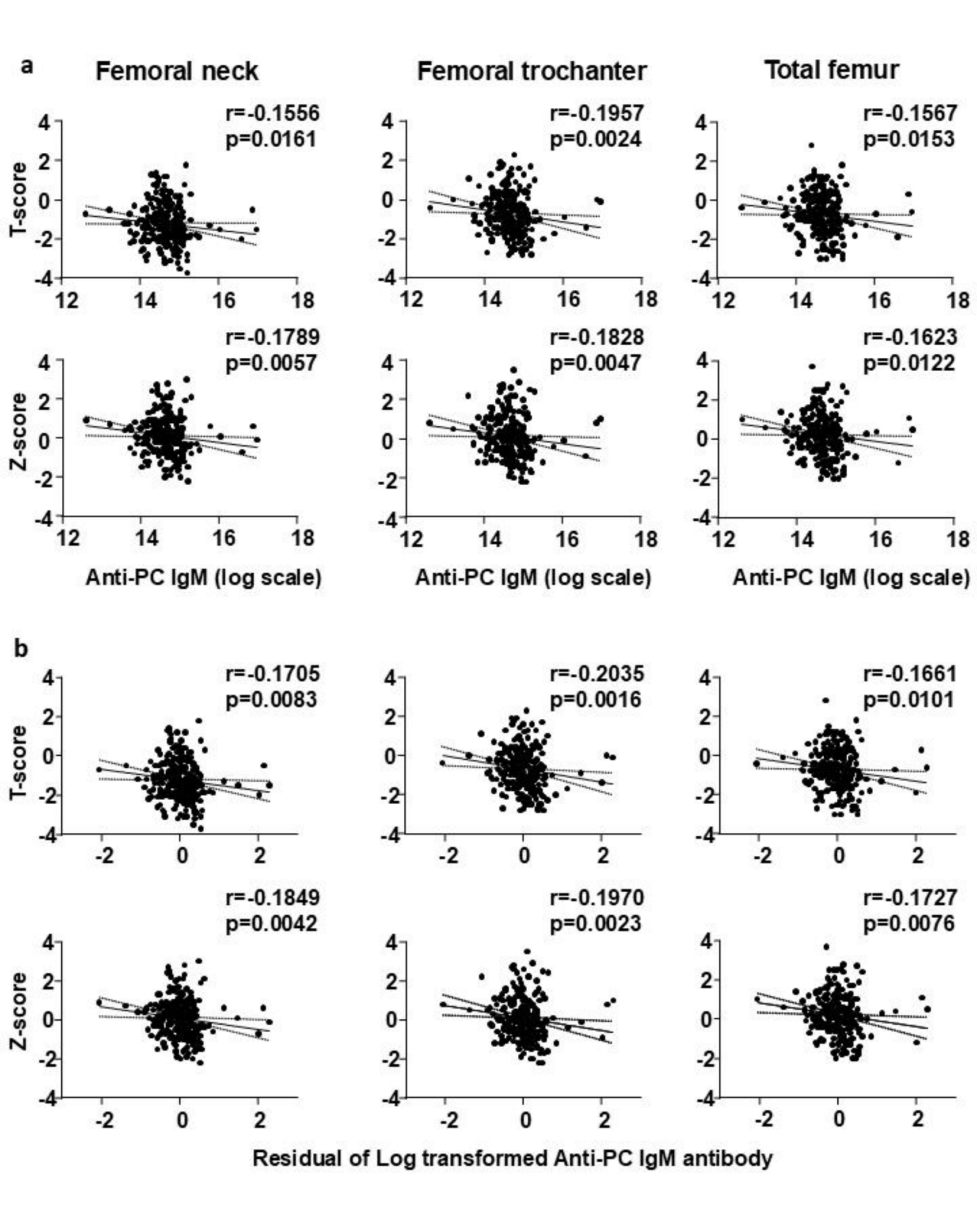
(**A**) Spearman correlation coefficients assessing the linear association between anti-PC IgM antibodies levels and DXA T-scores and Z scores at the femoral neck, femoral trochanter, and total femur. (**B**) Spearman correlation coefficients assessing the linear association between age-, sex-, and race-adjusted anti-PC IgM antibodies and DXA T-scores and Z-scores at the femoral neck, femoral trochanter, and total femur. A logarithmic transformation was applied to the anti-PC IgM measures prior to analysis. A linear regression on anti-PC IgM measures with covariates of age, sex, and race was used to produce the residuals.

**Fig. 3:**
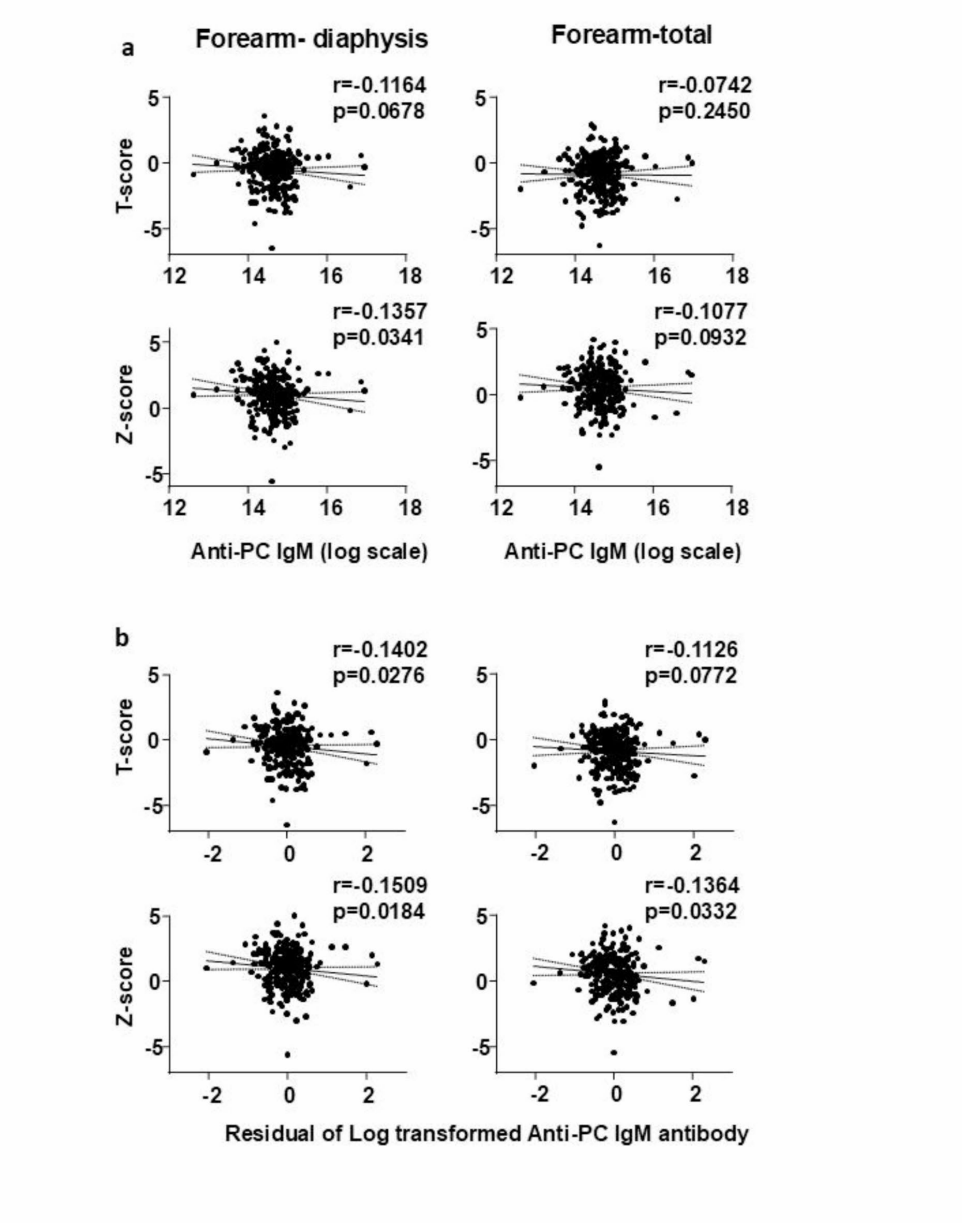
(**A**) Spearman correlation coefficients assessing the linear association between anti-PC IgM antibodies levels and DXA T-scores and Z scores at the forearm (diaphysis and total). (**B**) Spearman correlation coefficients assessing the linear association between age-, sex-, and race-adjusted anti-PC IgM antibodies and DXA T-scores and Z-scores at the forearm (diaphysis and total). A logarithmic transformation was applied to the anti-PC IgM measures prior to analysis. A linear regression on anti-PC IgM measures with covariates of age, sex, and race was used to produce the residuals.

The levels of anti-PC IgM were negatively correlated with the T-score and Z-score at the lumbar spine (Fig. 1a, Supplementary Tables S3, S5), and the correlation was maintained when the antibody levels were adjusted for age, race, and sex (Fig. 1b, Supplementary Tables S4, S6). Similar to the measurements at the spine, the levels of anti-PC IgM negatively correlated with the T-scores and Z-scores at the femoral neck, femoral trochanter, and total femur (Fig. 2a, Supplementary Tables S3, S5); and the correlation was maintained when the antibody levels were adjusted for age, race, and sex (Fig. 2b, Supplementary Tables S4, S6). At the forearm, the levels of the anti-PC IgM were negatively correlated only with the Z-score at the diaphysis but not with the T-scores at any site or Z-scores at the ultradistal and total regions (Fig. 3a, Supplementary Tables S3, S5). When the anti-PC IgM levels were adjusted for age, race, and sex there was a negative correlation with the T-score and Z-score at the diaphysis and the Z-score at the total forearm, but not with the T-score and Z-score at the ultradistal region (Fig. 3b, Supplementary Tables S4, S6).

### Comparison of anti-PC IgM levels in patients with normal BMD and osteopenia/osteoporosis

**Fig. 4:**
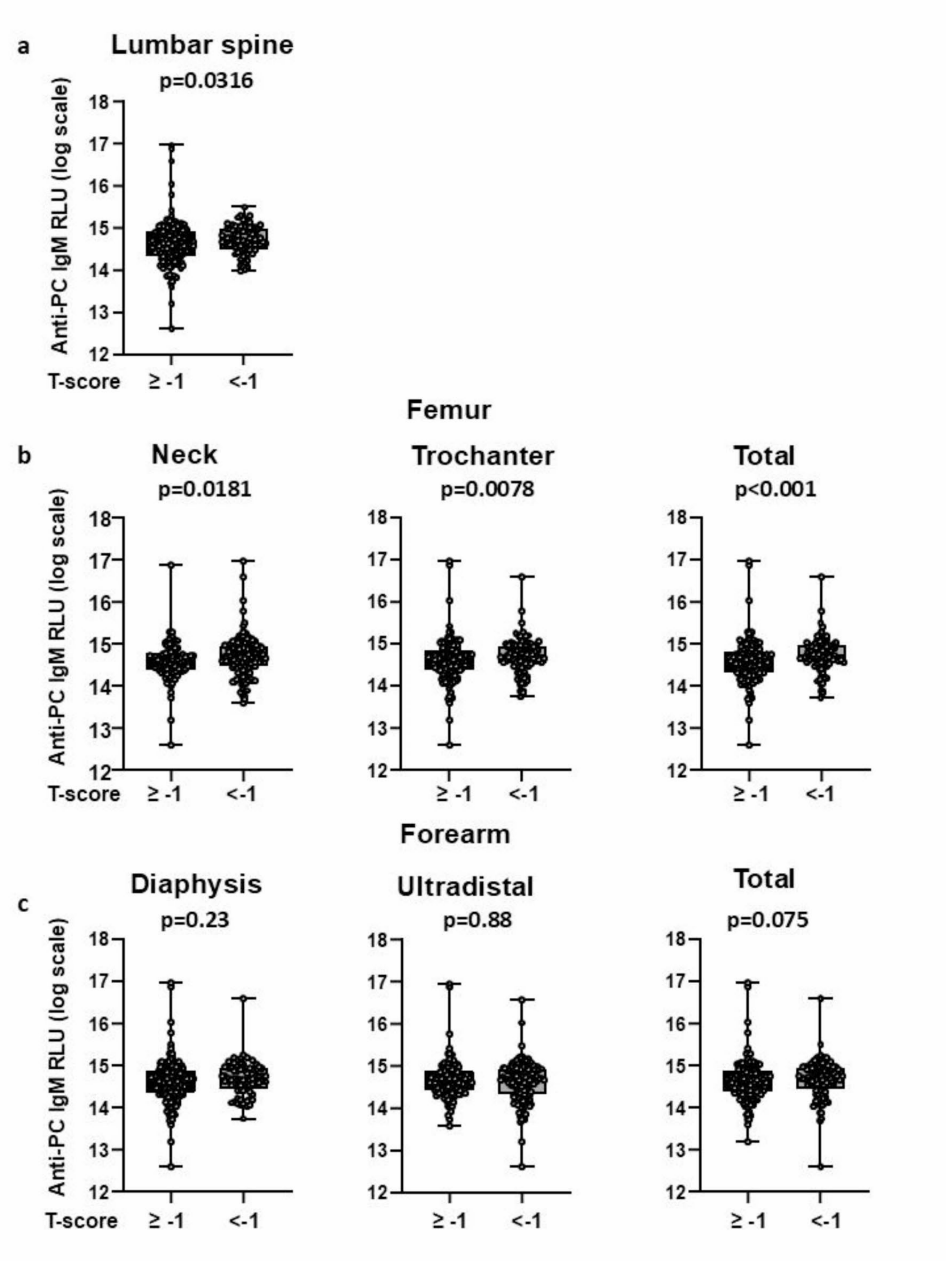
Anti- PC IgM levels (log transformed) in patients with normal T score (≥ − 1) or in patients with osteopenia and osteoporosis (T score < − 1) at the (**A**) lumbar spine, (**B**) femoral neck, femoral trochanter or total femur, and (**C**) forearm: diaphysis, ultradistal and total. Data analyzed by Wilcoxon Rank Sum test.

Consistent with the previous results, patients with T-scores < − 1 at the lumbar spine, femoral neck, femoral trochanter, and total femur had higher levels of anti-PC IgM than those with T-scores ≥ − 1 (*p* = 0.0316 at the lumbar spine, *p* = 0.0181 at the femoral neck, *p* = 0.0078 at the femoral trochanter and *p* < 0.0001 at the total femur) (Fig. 4). Collectively these results indicate that patients with higher levels of the anti-PC IgM had lower BMD.

We then analyzed the correlations between anti-PC IgM levels and T-scores separately in patients with T score < − 1 and T score ≥ − 1 at the different regions (Supplementary Tables S7 and S8). When we divided the patients in the two groups the number was considerably smaller and, therefore, we had inadequate statistical power. Nonetheless, the correlations became stronger at the lumbar spine in patients with T score < − 1, supporting the notion that patients with lower T-score have higher levels of anti-PC IgM.

### Anti-PC IgM levels as a marker of low BMD

To evaluate if the levels of anti-PC IgM could be used as a marker of low BMD, we performed a univariate linear regression analysis between the levels of anti-PC IgM and T-scores and Z-scores at the lumbar spine and femur (Supplementary Table S9). The percentage of the contribution of the antibody levels to the changes in T-score and Z-score varied between 1.9 and 2.4%. We also performed a receiver-operating characteristic curse (ROC) using the age, sex and race adjusted anti-PC IgM levels which indicated an AUC of 0.6274 (Supplementary Fig. S2).

## Discussion

In this cross-sectional study, we found that the levels of anti-PC IgM are negatively correlated with BMD at the spine, femur and, to a lesser extent, the forearm. This correlation was maintained after adjusting for age, race, and sex, and became stronger at the lumbar spine within the subgroup of patients diagnosed with osteopenia or osteoporosis. These findings suggest that higher levels of endogenous anti-PC IgM in patients with lower BMD may reflect exposure to higher levels of PC-OxPLs, which are known to affect bone mass. Although, this endogenous increase of anti-PC IgM does not confer protection against osteopenia or osteoporosis, anti-PC IgM levels could be used as a novel marker of osteoporosis.

PC-OxPLs are well recognized pathogens in atherosclerosis and nonalcoholic steatohepatitis^6,9^ causing protein degradation, apoptosis, necrosis and tissue fibrosis. PC-OxPLs bind to specific scavenger receptors and toll-like receptors on macrophages and endothelial cells, activate multiple pro-inflammatory responses, and increase the production of cytokines such as IL-1β, IL-6, IL-8 and TNF^1,30^. In vivo and in vitro data by us and others, have indicated that OxPLs decrease Wnt signaling—an essential factor for the differentiation and survival of osteoblasts^14,16,31–36^. Specifically, we found that in osteoblastic cells OxPLs decrease wingless-type MMTV integration site family member 10b (Wnt10b), which is one of the Wnt ligands that stimulates osteoblastogenesis though activation of the canonical Wnt signaling^37,38^. Consistent with these findings, blocking PC-OxPLs with E06 IgM prevented the negative effects of oxidized phospholipids on the proliferation, differentiation, and survival of osteoblastic cells in vitro^14^. Moreover, blocking PC-OxPLs with the E06-scFv transgene in vivo increased osteoblast number and bone formation rate, reduced osteoblast apoptosis and increased Wnt10b and Wnt signaling^16,39^.

Many investigators have evaluated the relationship between endogenous levels of anti-PC IgM and cardiovascular or autoimmune diseases in humans. In contrast to our findings, they found that low levels of anti-PC antibodies are associated with increased disease propensity. Specifically, low levels of anti-PC antibodies predict cardiovascular risk in men^40^, myocardial infarction^41^, peripheral vein graft failure^42^; and are associated with faster carotid intima media thickness progression^43^ and vascular remodeling in patients with coronary artery disease (CAD)^44^. In addition, low anti-PC IgM titers predict an increased risk for both fatal and non-fatal coronary events in patients with stable CAD^45^, major acute cardiovascular events and all-cause mortality in patients with acute coronary syndrome^46^ and hemodialysis^47^; and are an independent risk factor for the development of stroke in women^48^ and men^49^. Significantly lower levels of anti-PC IgM are found in patients with mixed connective tissue disease (MCTD), and are negatively correlated with cardiovascular diseases not only in patients with MCTD, but also in patients with rheumatoid arthritis, systemic lupus erythematous (SLE), and undifferentiated connective tissue disease^26,50^. In patients with SLE, in particular, the lowest tertile of anti-PC IgM is independently associated with the prevalence of atherosclerotic plaques^51^. Lower levels of anti-PC IgM, compared to healthy controls, are also found in patients with non-alcoholic fatty liver disease^52^ and Alzheimer’s dementia^53^. Consistent with this, multiple studies have established that high levels of anti-PC antibodies are protective against many age-associated diseases. Indeed, high levels of anti-PC IgM antibodies (as well as the levels of anti IgM anti-OxLDL and anti-malondialdehyde-modified low-density lipoprotein, MDA LDL) are predictive of the decreased rate of progression of carotid intima-media thickness in patients with hypertension^54,55^, associated with lower risk of acute myocardial infarction^56^, and protective against thromboembolic disease^57^. The levels of anti-PC IgM are significantly higher in patients with SLE who have low disease activity, less organ damage, and no cardiovascular events^58,59^. In contrast, a large prospective study in patients presenting with acute coronary syndrome showed that anti-PC IgM titers did not exhibit a significant relationship with cardiovascular outcomes such as myocardial infarction, stroke, or severe recurrent ischemia^29^.

It should be noted that this protective role of anti-PC antibodies is demonstrated in diseases characterized by chronic inflammation.

To the best of our knowledge, no other studies have identified an inverse relationship between anti-PC IgM and the severity of disease. Be that as it may, the pathogenetic mechanisms and thereby the immune response in atherosclerosis and osteoporosis are likely to be different: osteoporosis is the result of hormonal deficiencies and several aging-associated mechanisms termed “hallmarks of aging”^60^, whereas the predominant pathogenetic mechanism in cardiovascular diseases is inflammation.

Irrespective of disease protection or lack of thereof, all published studies indicate that the immune system responds to increased levels of PC-OxPLs with an increase in the endogenous levels of anti-PC IgM antibodies. Our findings are consistent with this observation, and suggest that anti-PC IgM levels may be used as biomarkers of enhanced exposure to PC-OxPLs.

PC-OxPLs, measured by ultrahigh performance liquid chromatography–tandem mass spectrometry, were shown by others to be elevated in LDL of patients with postmenopausal osteoporosis compared to healthy controls^61^. PC-OxPLs are generated during oxidation of LDL and carried by apolipoprotein B-100 containing lipoproteins, predominantly lipoprotein (a) [Lp(a)]. Postmenopausal women with elevated cholesterol (≥ 240 mg/dl), elevated LDLc (≥ 160 mg/dl) and or Lp(a) ≥ 25 mg/dl have lower BMD at the spine and femur^62^. Similarly, Lp(a) was negatively correlated with lumbar T-score in women with age ≥ 53 years^63^. In the post hoc analysis of the Women Heath Initiative, however, no significant association was found between the levels of plasma Lp(a) and low hip BMD T-score or hip fracture risk during a follow up of 13.8 years; albeit potential confounding factors could not be excluded^64^.

The univariate linear regression model shows that the contribution of anti-PC IgM to the change in T score and Z-score at the lumbar spine and femur is low. Nevertheless, it should be pointed out that it is similar to the contribution of 25-OH Vitamin D, PTH and alkaline phosphatase on the BMD change at the lumbar spine and femur in post-menopausal women^65^ as well as the contribution of physical activity, dietary calcium intake, smoking and ETOH intake on BMD in men^66^. Moreover, the results of the ROC analysis are similar to the ones found for alkaline phosphatase^67^ and β cross-linked C-telopeptide of type 1 collagen^68^ for BMD prediction in post-menopausal women. Likewise, our results are also similar to those found for osteocalcin and bone alkaline phosphatase in the prediction of BMD change after 1 year treatment with hormonal replacement therapy^69^. Be that as it may, the specificity and sensitivity of the current ELISA assay, is lower than the specificity and sensitivity of other markers of bone remodeling, in predicting BMD^70,71^. Future studies evaluating the levels of anti-PC IgM correlations with longitudinal changes of BMD will be necessary to assess the clinical usefulness of this marker.

Our study has several limitations. It is a cross-sectional study, and thus lacking the ability to detect potential causal relationships between changes in BMD and antibody levels. We measured only anti-PC IgM and not anti-PC IgG. However, the role of anti-PC IgG in diseases is less well established; only IgG1, but not IgG2, IgG3 or IgG4 have been shown to be associated with cardiovascular diseases^28^. We have enrolled a limited number of patients given the variability of the IgM levels detected; and less than 15% of the population had osteoporosis. Remarkably, and despite these seeming limitations, the “weak” correlations between the levels of the antibodies and the BMD parameters were consistently present in all three bone sites, in both the axial and appendicular skeleton, and for both the T- and Z-scores. Furthermore, when we restricted the analysis to patients with osteopenia and osteoporosis, the relationship became stronger at the spine, supporting the conclusion that anti-PC IgM levels may be biomarkers of bone loss. Further studies are required to evaluate the association of anti-PC IgM and bone mass in larger and older populations with higher prevalence of osteopenia and osteoporosis.

We did not measure the levels of oxidized phospholipids in our patients, and we did not have statistical power to calculate if the dyslipidemia is more prevalent in patients with low BMD because the patients without hyperlipidemia are only 10% of the entire group of participants. The LDL levels were similar between patients with and without a diagnosis of hyperlipidemia because all the patients with that diagnosis were treated with one of more therapies including statins, ezetimibe, PCSK9 inhibitors and lifestyle changes. Future studies are needed to examine the levels of PC-OxPLs in patients with lower bone mineral density using either mass spectrometry or the OxPL-ApoB assays recently optimized^30^.

The limitations notwithstanding, the clinical evidence reported herein, combined with compelling earlier preclinical evidence that neutralizing OxPLs promotes bone formation and prevents age-related bone loss in both male and female mice^14–16^, supports our overall working hypothesis that increasing anti-PC IgM antibodies pharmacologically may be a novel therapeutic approach to simultaneously treat two of the most common pathologies of old age in humans, osteoporosis and atherosclerosis.

## Materials and methods

### Study participants

We recruited 251 patients who had BMD measured by DXA at CAVHS, between February 2014 and September 2021. Initially, we identified Veterans who had a BMD done at CAVHS using data from the Corporate Data Warehouse through the VA Informatics and Computing Infrastructure (VINCI). The patients meeting criteria were contacted and enrolled in the study. With this modality we recruited 12 patients in 6 months and for those patients the average interval between the DXA and the blood collection was 3.5 ± 0.2 years. Because of the slow rate of recruitment, we changed the enrollment strategy and prospectively screened all patients who received a DXA at CAVHS every week. With this modality we recruited 239 patients from December 2019 until September 2021; patients received DXA and blood collection the same day or within a 2-week interval. The inclusion criteria for the study were: age above 18 and having a BMD by DXA done at CAVHS. We excluded patients with medical conditions or exposure to medications that could affect their BMD or the levels of anti-PC IgM antibodies. Exclusion criteria were as follows: treatment in the 5 years prior to the BMD with teriparatide, abaloparatide, bisphosphonates, denosumab, fluoride, strontium, or gallium nitrate; treatment in the 12 months prior to the BMD with calcitonin, calcitriol, selective estrogen receptor modulators (such as raloxifene or tamoxifen), tibolone, or anabolic steroids; hormonal treatment for gender dysphoria; treatment with anastrozole, GnRH agonists, antiandrogens in the 24 months prior to the BMD; treatment with anticonvulsants that affect Vitamin D metabolism (phenobarbital, phenytoin, carbamazepine, valproic acid, or primidone) at the time of BMD; treatment with chronic heparin within the 6 months prior to the BMD; daily treatment with oral corticosteroids within the 12 months prior to the BMD at a dose higher than 5 mg daily prednisone (or equivalent) or more than 3 steroid epidural injections in the year prior to BMD; history of metabolic bone disease other than osteoporosis (e.g. primary hyperparathyroidism, Paget’s disease, hypoparathyroidism); 25-vitamin D level < 20 ng/mL within 3 months from the BMD unless patient was started on replacement; history of hematological disorders (e.g. lymphoma, multiple myeloma, monoclonal gammopathy of undetermined significance, leukemia, anti-phospholipid syndrome); bone metastases to the femur or lumbar spine, or active malignancy on chemotherapy; history of malabsorptive syndromes (e.g. Crohn’s colitis, ulcerative colitis, cystic fibrosis, history of bariatric surgery); history of cirrhosis or splenectomy; history of human immunodeficiency virus or other immunodeficiencies, active immunotherapy against B-cell; history of solid organ or bone marrow transplants; history of chronic kidney disease stage 4 and 5 (glomerular filtration rate < 30 ml/min). At the study visit, vital signs were recorded (height, weight, body mass index and blood pressure measurements), and a blood sample was obtained at the CAVHS central lab. Patients’ serum and plasma samples were aliquoted, stored at − 80° and analyzed in batches. We also extracted from the electronic health record at CAVHS participants’ demographics, associated comorbidities, and laboratory measures of interest (calcium, parathyroid hormone, 25-OH vitamin D, phosphate, creatinine, albumin, estimated glomerular filtration rate, c-reactive protein, LDL and alkaline phosphatase).

### DXA scan

BMD was measured at the lumbar spine, proximal femur, and forearm by DXA Hologic Horizon A (S/N302674M, software version 1306.5). BMD was measured in g/cm^2^, and T-scores and Z-scores were obtained. Throughout the study, the least significant change for the BMD was 0.022 gr/cm^2^ for the total spine, 0.027 gr/cm^2^ for the total hip, 0.0104 gr/cm^2^ for the femoral neck, and 0.023 gr/cm^2^ for the 1/3 forearm. The quality control of the instrument was performed daily on lumbar spine phantom #103012, with CV of 0.333%.

### Anti-PC IgM measurements by ELISA

Human plasma IgM levels were determined via enzyme-linked immunosorbent assay (ELISA). U-bottom 96-well microtiter plates (catalog number 07-200-761, Fisher Scientific, Waltham, MA) were incubated overnight at 4 °C with 5 μg/ml PC(2)-BSA (catalog number PC-1011-10, Bioresearch Technologies, Petaluma, CA) diluted in phosphate-buffered saline (PBS) without calcium and magnesium (catalog number 21600010, ThermoFisher, Waltham, MA). After three washes with PBS, residual binding sites were blocked with StabilGuard Immunoassay Stabilizer (catalog number SG01-1000, Surmodics, Eden Prairie, MN) for 50 min at room temperature, followed by three additional washes with PBS. A standard curve was generated using native Human IgM protein (catalog number ab90348, AbCam, Waltham, MA). Standard curve points and plasma samples (diluted 1:200–1:800 in PBS) were added to appropriate wells and incubated for 90 min at room temperature, followed by three washes with PBS. All wells were then treated with goat-anti-Human IgM (mu chain) Antibody Alkaline Phosphatase Conjugated (Catalog Number 609-1507, Rockland, Limerick, PA) at a 1:500 dilution in SterilGuard buffer for 1 h at room temperature, followed by three washes with PBS. Plates were developed with Lumiphos 530 (Lumigen, Southfield, MI) for 60 min, and light emission was measured as relative light units (RLU) over 100 ms using a plate luminometer. Only values within the linear range of the standard curves were utilized**.** The results of the RLU were adjusted to the RLU for the standard curve in every plate. The assay has an in inter-assay coefficient of variation (CV) of 14% and intra-assay CV of 3.42%.

### Statistical analysis

Continuous variables were summarized by mean and standard deviation (SD) and median (interquartile range). Categorical variables were summarized by percentages and counts. Anti-PC IgM values were adjusted for age, sex, and race by analyzing the residuals from a linear regression on anti-PC IgM using those factors as covariates. Two-sample tests were used to test the levels of anti-PC IgM values at various sites based on T-scores greater than or equal to − 1 vs. less than − 1. Dependent on the distribution of the values either an equal variances t-test, an unequal variances t-test, or a Wilcoxon Rank Sum test for non-normal data was applied. Spearman correlations were used due to non-normality of some variables. Strength of relationships between BMD values and antibody levels was further assessed by the r-squared of univariate logistic regressions. Univariate logistic regression was fit to produce a receiver-operating characteristic curve (ROC) for antibody levels predicting osteoporosis diagnosis. All analyses were performed with SAS software, version 9.4.

### Study approval

For study participation, patients signed an informed consent. This study was approved by the local Institutional Review Board at CAVHS. All experiments were performed in accordance with the relevant guidelines and regulations.

## Electronic supplementary material

Below is the link to the electronic supplementary material.

Supplementary Material 1

## Data Availability

The data that support the findings of this study are not openly available due to reasons of sensitivity and are available from the corresponding author upon reasonable request. Data are located in controlled access data storage at the Central Arkansas Veterans HealthCare System.
