# Supplementary material for: Plasma levels of anti phosphocholine IgM antibodies are negatively correlated with bone mineral density in humans
Source: Sci Rep. 2025 Jan 15;15:2109. doi: 10.1038/s41598-025-85624-9 (PMC11735633; doi:10.1038/s41598-025-85624-9)
Supplement: Supplementary file 1 — Supplementary Material 1 [file 41598_2025_85624_MOESM1_ESM.pdf]

Plasma Levels of Anti-Phosphocholine IgM Antibodies are Negatively Correlated with Bone Mineral Density in Humans.

Michela Palmieri[1], Spyridoula Maraka[1,2], Horace J Spencer[3†], Jeff D. Thostenson[3], Katherine Dishongh[2], Micheal Knox[2], Betty Ussery[2], Jesse Byrd[2], Jacqueline K. Kuipers[2], Sanaz Abedzadeh-Anaraki[4], Chitharanjan Duvoor[5], Yuanjie Mao[6], Lakshmi Menon[1], James S. Williams[2], Stavros C. Manolagas[1], Robert L Jilka[1], and Elena Ambrogini[1,2]*

Supplementary Figure S1

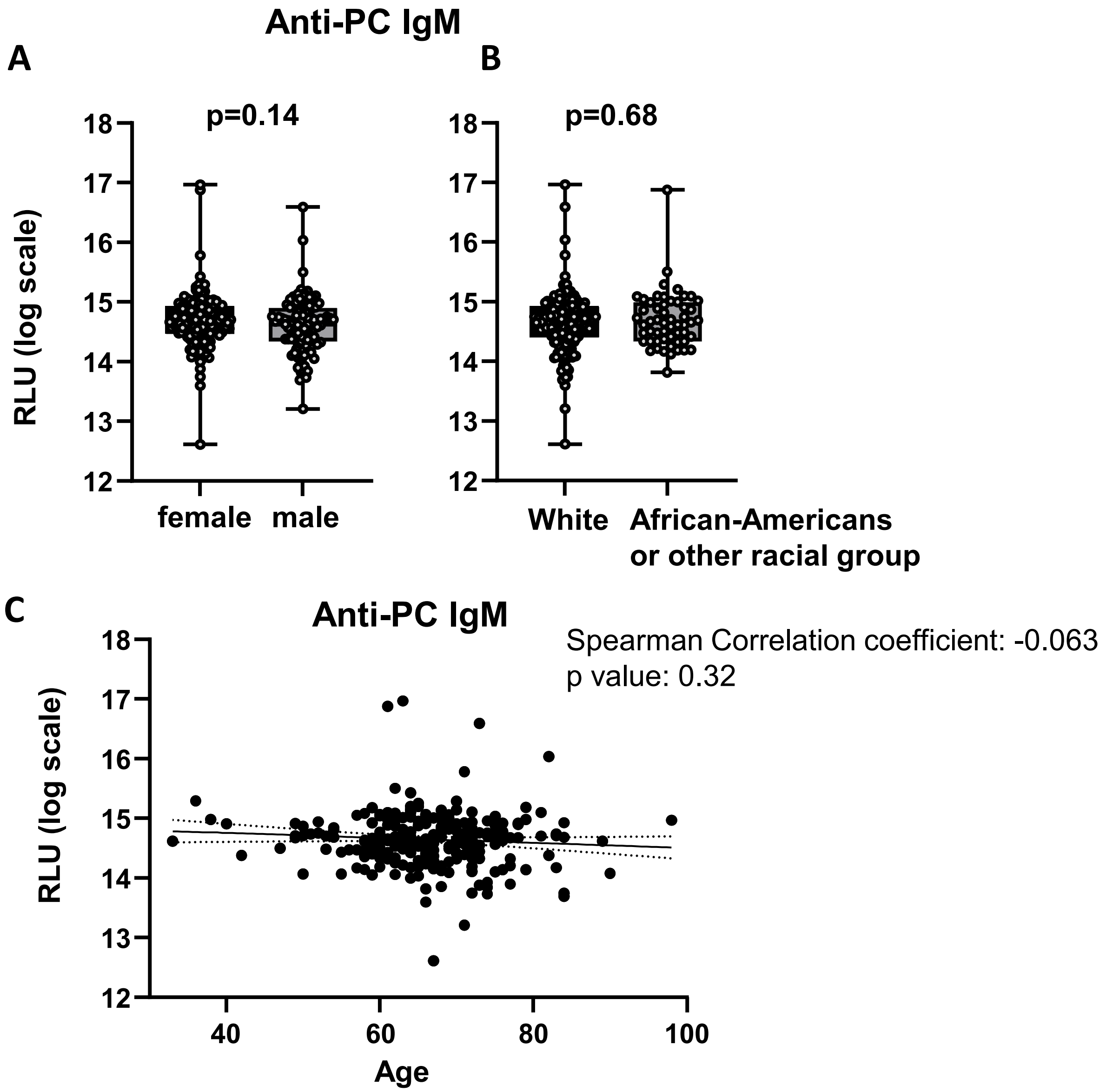

Supplementary Figure S1: A) Anti- PC IgM levels (log transformed) in females and males, and White vs African Americans or other racial groups. Data analyzed by Wilcoxon Rank Sum test. B) Spearman correlation coefficient assessing the linear association between anti-PC IgM antibodies and age. A logarithmic transformation was applied to the IgM anti-PC measures prior to analysis.

Supplementary Figure S2

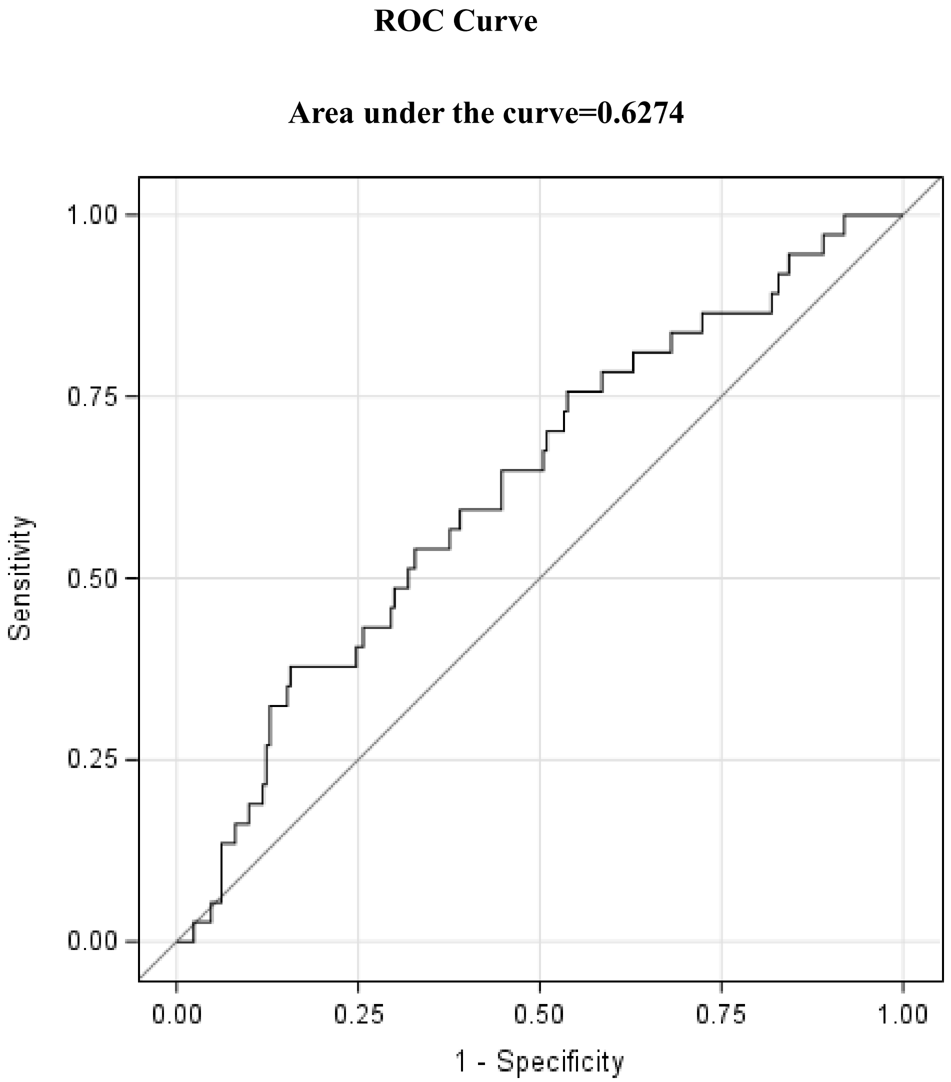

Supplementary Figure S2: Receiver-operating characteristic curve of the age, sex and race adjusted anti-PC IgM levels**.** The area under the curve provides a measure of predictive power for the diagnosis of low bone mass.

Supplementary Table S1. Summary of Laboratory Measures.

Variable	N	Mean (SD)	Median (IQR)	Normal range
Calcium (mg/dL)	246	9.3 (0.3)	9.2 (0.5)	8.3-10.3
Ionized Calcium (mmol/L)	82	1.17 (0.05)	1.16 (0.07)	1.13-1.32
PTH (pg/ml)	91	46.3 (18.6)	41.6 (26.4)	12-88
25-OH-Vitamin D (ng/ml)	191	39.3 (16.8)	36.9 (19.8)	30-100
Phosphate (mg/dL)	101	3.6 (0.6)	3.6 (0.7)	2.5-4.9
Creatinine (mg/dL)	247	0.97 (0.26)	0.90 (0.30)	0.6-1.3
Albumin (g/dL)	244	3.9 (0.3)	4.0 (0.4)	3.4-5.0
EGFR (ml/min/1.73 m^2)	246	57.7 (5.5)	60.1 (0.0)	>60
C-reactive protein (mg/L)	240	4.9 (7.9)	2.3 (4.7)	0-6.0
IgG (mg/dL)	243	1068.7 (289.3)	1032.5 (359.6)	610.3-1616.0
IgM (mg/dL)	243	92.6 (60.1)	77.9 (51.7)	35-242
IgM Anti-PC Adjusted RLU	247	2634330.56 (2217721.07)	23332357.11 (1209001.39)	

IQR: interquartile range
RLU: relative light units

Supplementary Table S2. Spearman correlation coefficients between anti-PC IgM and total IgM

	N	Spearman Correlation Coefficient	P-value
IgM	243	0.58	<0.0001

Supplementary Table S3. Spearman correlation coefficients between anti-PC IgM and DEXA T-scores

Region	N	Spearman Correlation Coefficient	P-value
Lumbar L1-L4	221	-0.1588	**0.0181**
Femoral Neck	239	-0.1556	**0.0161**
Femoral Trochanter	239	-0.1957	**0.0024**
Total Femur	239	-0.1567	**0.0153**
Forearm- Diaphysis	247	-0.1164	0.0678
Forearm- Ultradistal	247	-0.0028	0.9648
Forearm- Total	247	-0.0742	0.2450

Spearman correlation coefficients assessing the linear association between anti-PC IgM antibodies levels and DXA T-scores for each anatomical region. A logarithmic transformation was applied to the anti-PC IgM measures prior to analysis.

Supplementary Table S4. Spearman correlation coefficients between age, sex and race adjusted anti-PC IgM and DEXA T-scores

Region	N	Spearman Correlation Coefficient	P-value
Lumbar L1-L4	221	-0.1409	**0.0363**
Femoral Neck	239	-0.1705	**0.0083**
Femoral Trochanter	239	-0.2035	**0.0016**
Total Femur	239	-0.1661	**0.0101**
Forearm- Diaphysis	247	-0.1402	**0.0276**
Forearm- Ultradistal	247	-0.0524	0.4127
Forearm- Total	247	-0.1126	0.0772

Spearman correlation coefficients assessing the linear association between Age, Sex, and Race adjusted anti-PC IgM antibodies and DEXA T-scores for each anatomical region. A logarithmic transformation was applied to the anti-PC IgM measures prior to analysis. A linear regression on anti-PC IgM measures with covariates of Age, Sex, and Race was used to produce the residuals.

Supplementary Table S5. Spearman correlation coefficients between anti-PC IgM and DEXA Z-scores

Region	N	Spearman Correlation Coefficient	P-value
Lumbar L1-L4	221	-0.1591	**0.0179**
Femoral Neck	238	-0.1789	**0.0057**
Femoral Trochanter	238	-0.1828	**0.0047**
Total Femur	238	-0.1623	**0.0122**
Forearm- Diaphysis	244	-0.1357	**0.0341**
Forearm- Ultradistal	244	-0.0291	0.6505
Forearm- Total	244	-0.1077	0.0932

Spearman correlation coefficients assessing the linear association between anti-PC IgM antibodies levels and DXA Z-scores for each anatomical region. A logarithmic transformation was applied to the anti-PC IgM measures prior to analysis.

Supplementary Table S6. Spearman correlation coefficients between age, sex and race adjusted anti-PC IgM and DEXA Z-scores

Region	N	Spearman Correlation Coefficient	P-value
Lumbar L1-L4	221	-0.1580	**0.0188**
Femoral Neck	238	-0.1849	**0.0042**
Femoral Trochanter	238	-0.1970	**0.0023**
Total Femur	238	-0.1727	**0.0076**
Forearm- Diaphysis	244	-0.1509	**0.0184**
Forearm- Ultradistal	244	-0.0547	0.3946
Forearm- Total	244	-0.1364	**0.0332**

Spearman correlation coefficients assessing the linear association between Age, Sex, and Race adjusted anti-PC IgM antibodies and DEXA S-scores for each anatomical region. A logarithmic transformation was applied to the anti-PC IgM measures prior to analysis. A linear regression on anti-PC IgM measures with covariates of Age, Sex, and Race was used to produce the residuals.

Supplementary Table S7. Spearman correlation coefficients between anti-PC IgM and DEXA T-scores

Region	Spearman Correlation Coefficient –participants with T score <-1 (p-value)		Spearman Correlation Coefficient – participants with T score ≥1 (p-value)	
Lumbar L1-L4	n=76	**-0.2576** (**0.0247**)	n=145	-0.0282 (0.7361)
Femoral Neck	n=145	-0.1071 (0.1999)	n=94	0.1060 (0.3094)
Femoral Trochanter	n=96	-0.1851 (0.0711)	n=143	-0.0434 (0.6065)
Total Femur	n=93	-0.0743 (0.4793)	n=146	0.1100 (0.1862)

Spearman correlation coefficients assessing the linear association between anti-PC IgM antibodies levels and DXA T-scores for each anatomical region in participants with T-score <-1 and T score ≥-1. A logarithmic transformation was applied to the anti-PC IgM measures prior to analysis.

Supplementary Table S8. Spearman correlation coefficients between age, sex and race adjusted anti-PC IgM and DEXA T-scores

Region	Spearman Correlation Coefficient –participants with T score <-1 (p-value)		Spearman Correlation Coefficient –participants with T score ≥1 (p-value)	
Lumbar L1-L4	n=76	**-0.2761 (0.0158)**	n=145	-0.0102 (0.9035)
Femoral Neck	n=145	-0.1370 (0.1002)	n=94	0.0933 (0.3713)
Femoral Trochanter	n=96	-0.1882 (0.0663)	n=143	-0.0356 (0.6730)
Total Femur	n=93	-0.0952 (0.3641)	n=146	0.1000 (0.2297)

Spearman correlation coefficients assessing the linear association between Age, Sex, and Race adjusted anti-PC IgM antibodies and DEXA T-scores for each anatomical region in participants with T-score <-1 and T score ≥-1. A logarithmic transformation was applied to the anti-PC IgM measures prior to analysis. A linear regression on anti-PC IgM measures with covariates of Age, Sex, and Race was used to produce the residuals.

Supplementary Table S9. Univariate Linear regression model

Anti-PC IgM and DEXA T-scores

**Region T-score**	**R^2**	**p-value**
Lumbar L1-L4	0.0075	0.1991
Femoral Neck	0.0124	0.0863
Femoral Trochanter	0.0193	**0.0317**
Total Femur	0.0142	0.062

Age, sex and race adjusted anti-PC IgM Anti-PC IgM and DEXA T-scores

**Region T-score**	**R^2**	**p-value**
Lumbar L1-L4	0.0087	0.1665
Femoral Neck	0.0162	**0.0496**
Femoral Trochanter	0.0241	**0.0162**
Total Femur	0.0180	**0.0383**

Anti-PC IgM and DEXA Z-scores

**Region Z-score**	**R^2**	**p-value**
Lumbar L1-L4	0.0073	0.2050
Femoral Neck	0.0165	**0.0475**
Femoral Trochanter	0.0172	**0.0432**
Total Femur	0.0162	**0.0499**

Age, sex and race adjusted anti-PC IgM Anti-PC IgM and DEXA Z-scores

**Region Z-score**	**R^2**	**p-value**
Lumbar L1-L4	0.0120	0.1046
Femoral Neck	0.0202	**0.0283**
Femoral Trochanter	0.0249	**0.0148**
Total Femur	0.0221	**0.0218**

Univariate Linear Regression models were fit on the log-transformed data to calculate the R^2 and p values.
